# Alterations of Prefrontal-Posterior Information Processing Patterns in Autism Spectrum Disorders

**DOI:** 10.3389/fnins.2021.768219

**Published:** 2022-01-31

**Authors:** Hai-Chen Zhao, Rui Lv, Guang-Yu Zhang, Le-Min He, Xiao-Tao Cai, Qiang Sun, Chun-Yan Yan, Xiang-Yuan Bao, Xin-Yue Lv, Bin Fu

**Affiliations:** Department of Radiology, Shandong First Medical University & Shandong Academy of Medical Sciences, Tai'an, China

**Keywords:** autism spectrum disorders, entropy connectivity, predictive coding theory, information processing, rest-state fMRI

## Abstract

Autism spectrum disorder (ASD) is a heterogeneous disorder characterized by different levels of repetitive and stereotypic behavior as well as deficits in social interaction and communication. In this current study, we explored the changes in cerebral neural activities in ASD. The purpose of this study is to investigate whether there exists a dysfunction of interactive information processing between the prefrontal cortex and posterior brain regions in ASD. We investigated the atypical connectivity and information flow between the prefrontal cortex and posterior brain regions in ASD utilizing the entropy connectivity (a kind of directional connectivity) method. Eighty-nine patients with ASD and 94 typical developing (TD) teenagers participated in this study. Two-sample t-tests revealed weakened interactive entropy connectivity between the prefrontal cortex and posterior brain regions. This result indicates that there exists interactive prefrontal-posterior underconnectivity in ASD, and this disorder might lead to less prior knowledge being used and updated. Our proposals highlighted that aforementioned atypical change might accelerate the deoptimization of brain networks in ASD.

## Introduction

Autism spectrum disorder (ASD) is a neurodevelopmental disorder. According to the data from the Centers for Disease Control and Prevention (CDC) reported in 2020, one out of every 54 children is diagnosed with ASD. The main symptoms of ASD are deficits in social interaction and stereotypical or repetitive behavior (Maenner et al., 2020).

Previous studies have found neural underpinnings of ASD to be heterogeneous. Quantifying the size of the brains of autistic patients revealed early gray matter and white matter hyperplasia (Courchesne et al., 2001) but Lee et al. found that autistic children without megalencephaly had rather comparable gray and white matter development to non-autistic children (Lee et al., 2020), which may suggest cortical development as a heterogeneous condition for ASD. Neuroimaging studies found that ASDs presented atypical functional connectivity patterns in brains, such as underconnectivity (Just et al., 2004; Schipul et al., 2011; Starck et al., 2013), overconnectivity (Delmonte et al., 2013; Li et al., 2020; Seghatol-Eslami et al., 2020) and mixed connectivity (Monk et al., 2009; Chen et al., 2018; Oldehinkel et al., 2019). These altered functional connections affect the functions of multisensory, social communication, and high-level cognitive activities. However, it is not entirely clear how abnormal functional connectivity affects the clinical features of ASD. Some researchers have attempted to use directional functional connectivity to explore the mechanism by which the changes of brain connectivity leading to atypical symptoms. Weaker effective connectivity from the ventral attention network to the salience-executive network in adolescents with IQs in the normal range was also found by using the Granger causality method (Bernas et al., 2018). In addition, previous studies also detected abnormalities within language networks of ASD which depend on directional connectivity pattern from the precuneus *via* caudate nucleus to interior frontal gyrus rather than the connectivity pattern from the interior frontal gyrus via caudate nucleus to the precuneus by using dynamic causal modeling (DCM) (Radulescu et al., 2013) and verified underconnectivity between brain regions of ASD utilizing transfer entropy and graph theory (Ejman et al., 2017).

Some studies have reported atypical alterations in the structural properties of the prefrontal cortex in ASDs, such as increased gray matter volume in the left frontal and right medial prefrontal cortex (Deramus and Kana, 2015), atypical developmental trajectory in the volume of the dorsolateral prefrontal cortex (Carper and Courchesne, 2005), developmental abnormality of minicolumns in the dorsal and orbital frontal cortices (Buxhoeveden et al., 2006), and weakened asymmetries in the cortical thickness and surface area of the medial orbitofrontal cortex (Postema et al., 2019). It is worth noting that the prefrontal lobe plays a key role in ASD (Damasio and Maurer, 1978; Mundy, 2003). ASDs with dysfunction of the prefrontal cortex display abnormalities in some cognitive functions, such as working memory (Koshino et al., 2005; Vogan et al., 2018), cognitive control (Solomon et al., 2014; Lukito et al., 2020), mentalizing (Spengler et al., 2010), effortful control (Krishnamurthy et al., 2020), and self-referential processing (Burrows et al., 2016; Hashimoto et al., 2017). Additionally, ASDs also presented changes in structure and functions in some posterior brain regions including the parietal, occipital, temporal lobes. For instance, some researchers found the atypical changes in the middle temporal gyrus (Pappaianni et al., 2018), fusiform gyrus (Kuno-Fujita et al., 2020), interior parietal lobes (May and Kana, 2020) and sensorimotor cortex (Sapey-Triomphe et al., 2019).

The predictive coding theory suggested that higher hierarchies are involved in the storage and application of the prior knowledge and lower hierarchies are related to the information integration from body and environment. The higher hierarchies initiate modulation signals to actively infer the state of body and world and lower hierarchies also generate feedback input to revise the prior knowledge (Friston, 2010; Smith et al., 2017). This functional interaction is similar to the information communication between the prefrontal cortex and other brain regions. For instance, Miller (2000) and Duncan (2001) had reviewed much evidence that the prefrontal cortex can broadcast the signals from prior knowledge to other neocortical regions (e.g., the inferior temporal cortex and posterior parietal cortex) to modulate the information in them. Furthermore, the ecological model of the prefrontal cortex proposed that the prefrontal cortex implements niche construction through facilitating the construction of rules and norms that guide learning and behavior, and biasing processing in posterior neural regions to align with currently relevant rules and norms. In turn, the posterior brain regions projects the information from body and environment to the prefrontal cortex to drive it to adapt the changes of information sampling (Werchan and Amso, 2017). Underconnectivity between the frontal and posterior regions in ASD has been reported in previous studies (Fulvia et al., 2002; Kana et al., 2009). However, it is unclear whether there exists an alteration of interactive information flow between the prefrontal cortex and posterior brain regions and its potential effect. We hypothesized that the changes of interactive information flow might influence the deoptimization of the autistic brain networks. In this present study, we explored the hypothesis by using entropy connectivity (a kind of directional connectivity, the entropy connectivity between two brain areas reflects the direction of information flow from one brain area to the other). Our objective is to research the changes of the prefrontal-posterior information processing patterns and its potential influences.

## Experimental Procedures

### Subjects

Eighty-nine patients (age, 15–20 years; mean = 15.1 ± 5.1 years) and ninety-four well-matched teenagers (age, 16–19 years, mean = 16.0 ± 4.2 years) participated in this study. All data of these participants come from the Autism Brain Imaging Data Exchange (ABIDE), including PITT, SDSU, UM, YALE, CMU, NYU, STANFORD, UCLA, CALTECH, USM, and LEUVEN (Di Martino et al., 2014). All procedures performed in studies involving human participants were in accordance with the ethical standards of the Institutional Ethics Committee of Shandong First Medical University and with the 1964 Helsinki declaration and its later amendments or comparable ethical standards (R202104130149).

### Functional Magnetic Resonance Imaging Data

Data used in our study come from eleven sites. Full details are shown in Table 1. For more information about scanner types and parameters, please visit the website (http://fcon_1000.projects.nitrc.org/indi/abide/abide_I.html).

**Table 1:** Summary table of the train and test datasets.

**Acquisition site**	**Nr. ASD's**	**Nr. TD's**	**Nr. subjects**	**Age, ASD**	**Age, TD**	**Scanner**
CALTECH	3	1	4	20.9 ± 1.3	20.8	SIEMENS MAGNETOM TrioTim syngo MR B17
PITT	10	3	13	15.1 ± 4.9	22.2 ± 7.3	SIEMENS MAGNETOM Allegra syngo MR A30
SDSU	8	13	21	14.9 ± 2.0	14.3 ± 1.6	GE 3T MR750
UM	18	15	33	14.1 ± 2.5	14.2 ± 2.7	3 Tesla GE Signa
YALE	8	10	18	12.9 ± 3.0	14.5 ± 1.2	SIEMENS MAGNETOM TrioTim syngo MR B17
CMU	3	0	3	27.0 ± 6.9	0	SIEMENS MAGNETOM Verio syngo MR B17
NYU	9	19	28	19.1 ± 9.5	15.1 ± 5.0	SIEMENS MAGNETOM Allegra syngo MR 2004A
STANFORD	2	0	2	11.4 ± 0.7	0	GE SIGNA 3T
UCLA	28	12	40	13.4 ± 2.6	13.7 ± 1.2	SIEMENS MAGNETOM TrioTim syngo MR B15
USM	0	10	10	0	17.9 ± 3.5	SIEMENS MAGNETOM TrioTim syngo MR B17
LEUVEN	0	11	11	0	22.1 ± 1.8	LEUVEN-1: PHILIPS INTERA 3T

Firstly, data were preprocessed by using the DPARSF (Processing Assistant for Resting-State fMRI) software (http://preprocessed-connectomes-project.org/abide/Pipelines.html) and this preprocessing steps included dropping first 4 volumes (scrubbing), slice timing, motion realignment, registration [a transform from original to template (MNI152) space was calculated for each dataset from a combination of functional-to-anatomical and anatomical-to-template transforms. The anatomical-to-template transforms were calculated using a two step procedure that involves (one or more) linear transform that is later refined with a very high dimensional non-linear transform], Gaussian smoothing (6 × 6 × 6 mm), detrend, nuisance regression (including the removal of realignment parameters, mean white matter and cerebrospinal fluid signals), band-pass temporal filtering (0.01–0.1 HZ), Then, all preprocessed fMRI data were further processed by using the virtual digital brain software package VDB1.7 (https://www.nitrc.org/projects/vdb/). The steps are described as follows: (1) calculate causal connectivity between BAs (Brodmann's areas); (2) statistical analysis; (3) result display.

### Entropy Connectivity

The Brodmann area (BA) atlas was normalized as a standard MNI brain template. Each BA is regarded as the seed of entropy connectivity. All brain regions in the BA template were selected as seed regions, however, only those that presented significant changes in the prefrontal cortex were analyzed in this study. The index of BA is defined in Table 2. Functional connectivity reflects statistical correlations between brain regions. To describe the direction of the functional connectivity, in the present study, we adopted an entropy connectivity method that has been used in a previous study (Zhang et al., 2016). Entropy connectivity between two brain areas reflects the direction of information flow from one brain area to the other. The steps of entropy connectivity are described as follows: (1) obtain the BOLD signal of a BA X and a BA Y from the time series (t_1_ → t_n_); (2) obtain the change directions of the BOLD signal of BA X in a certain time interval Δt_1_ and BA Y in the next intermittent time interval Δt_2_; (3) compare the direction of changes and repeat the second step to obtain the probability of the same and opposite changes, respectively, which were observed throughout the time series; (4) if Bayesian probability acquired in the third step [P(Y/X)] > 0.5 simultaneously Pearson correlation coefficient r > 0, it is defined the synchronous entropy connectivity from the BA X to BA Y. Similarly, if the BOLD signal changes in the opposite direction and r < 0, it is defined as an asynchronous entropy connectivity from the BA Y to BA X.

**Table 2:** Indexes and corresponding brain regions.

**Indexes**	**Brodmann area**	**Corresponding brain regions in AAL template**
BA 1L	Left primary somatosensory cortex	Left precentral gyrus
BA 2L	Left primary somatosensory cortex	Left precentral gyrus
BA 2R	Right primary somatosensory cortex	Right precentral gyrus
BA 7L	Left somatosensory association cortex	Left somatosensory association cortex
BA 7R	Right somatosensory association cortex	Right superior parietal lobule
BA 8R	Right dorsal frontal cortex	Right middle frontal gyrus
BA 9L	Left dorsolateral prefrontal cortex	Left middle frontal gyrus/medial superior frontal gyrus
BA 9R	Right dorsolateral prefrontal cortex	Right middle frontal gyrus/medial superior frontal gyrus
BA 10L	Left anterior prefrontal cortex	Left orbital superior frontal gyrus
BA 11L	Left orbitofrontal cortex	Left rectus
BA 17R	Right primary visual cortex	Right calcarine
BA 20R	Right inferior temporal gyrus	Right inferior temporal gyrus
BA 21L	Left middle temporal gyrus	Left middle temporal gyrus
BA 21R	Right middle temporal gyrus	Right middle temporal gyrus
BA 24L	Left ventral anterior cingulate cortex	Left anterior/middle cingulum
BA 24R	Right ventral anterior cingulate cortex	Right anterior/middle cingulum
BA 32L	Left dorsal anterior cingulate cortex	Left anterior cingulum
BA 33L	Left anterior cingulate cortex	N/A
BA 39L	Left angular gyrus	Left angular gyrus
BA 39R	Right angular gyrus	Right angular gyrus
BA 40L	Left supramarginal gyrus	Left supramarginal gyrus
BA 43L	Left subcentral area	Left postcentral gyrus/Rolandic operculum
BA 45L	Left IFC pars triangularis	Left inferior frontal gyrus triangle
BA 46R	Right dorsolateral prefrontal cortex	Right inferior frontal gyrus triangle
BA 47R	Right inferior prefrontal gyrus	Right orbital inferior frontal gyrus

Entropy connectivity describes the interregional causality and information flow. The entropy connectivity of the synchronous change of BOLD signals in two brain regions is also called synchronous entropy connectivity, which indicates cooperative relation between two brain regions, that is, these two brain areas work with consistent steps. Similarly, the entropy connectivity of the asynchronous change is also called asynchronous entropy connectivity, which reflects inconsistent work pattern between two brain regions, that is, they work with opposite steps. Synchronous output entropy connectivity indicates the change of BOLD signal in one brain will drive that in the other with the same change trend, and asynchronous output entropy connectivity indicates the change of BOLD signal in one brain will drive that in the other with the opposite change trend. Synchronous input entropy connectivity denotes the change of BOLD signal in one brain is driven by that in the other with the same change trend, and asynchronous input entropy connectivity denotes the change of BOLD signal in one brain is driven by that in the other with the opposite change trend. Increased synchronous entropy connectivity indicates enhanced cooperative work pattern between brain regions; decreased synchronous entropy connectivity indicates reduced cooperative work pattern between brain regions. In contrast, increased asynchronous entropy connectivity indicates enhanced opposite work pattern between brain regions; decreased asynchronous entropy connectivity indicates reduced opposite work pattern between brain regions. A detailed description can be found in a study by Zhang et al. (2016).

### Positive Reproducible Test

For any given sample size, the probability of false positives and false negatives is a zero-sum game. To obtain a trade-off between the probability of false positives and false negatives, we used the positive reproducible test (PRT) method (Zhang et al., 2021) to correct statistical results. Decreased type I errors will lead to increased type II errors, the PRT method can obtain a trade-off between the false positive and negative probabilities by randomly selecting samples and repeating this test. This method can obtain low false negative probability and few type I errors through selecting high positive reproducible rate in the statistical hypothesis testing. In order to illustrate this issue, we performed the comparison between the PRT correction and the uncorrected results for the synchronous entropy connectivity of the whole brain.

Firstly, we executed a statistical test for synchronous entropy connectivity and selected a high probability of false positives (*p* = 0.05), which is responding to a low probability of false negatives, and then performed the steps below. (a) Execute the statistical test 1,000 times with 70 subjects selected randomly in every group under a certain positive reproducible rate. (b) Repeat step (a) 100 times with the positive reproducible rate selected from 1 to 100% with an interval of 1%. (c) Calculate the mean of those *p* values responding to every positive reproducible rate for every synchronous entropy connectivity. (d) Obtain the number of positive results for every positive reproducible rate and averaged *p* value. (e) Calculate the probability of false negatives. Let *M* and *m* denote the total number of independent experiments and true positive results in the statistical hypothesis testing respectively. *m*_*i*_ and *p*_*i*_ denote the number of positive results and the probability of false positives in the *i-th* test respectively. Then the probability of false negatives *P*_*fn*_(*i*) is written as $P_{fn}\left(i\right)=\frac{\left[m-m_{i}\times \left(1-p_{i}\right)\right]}{M}$, where *i* = 1, 2, ⋯ , 100.

**Figure 1:**
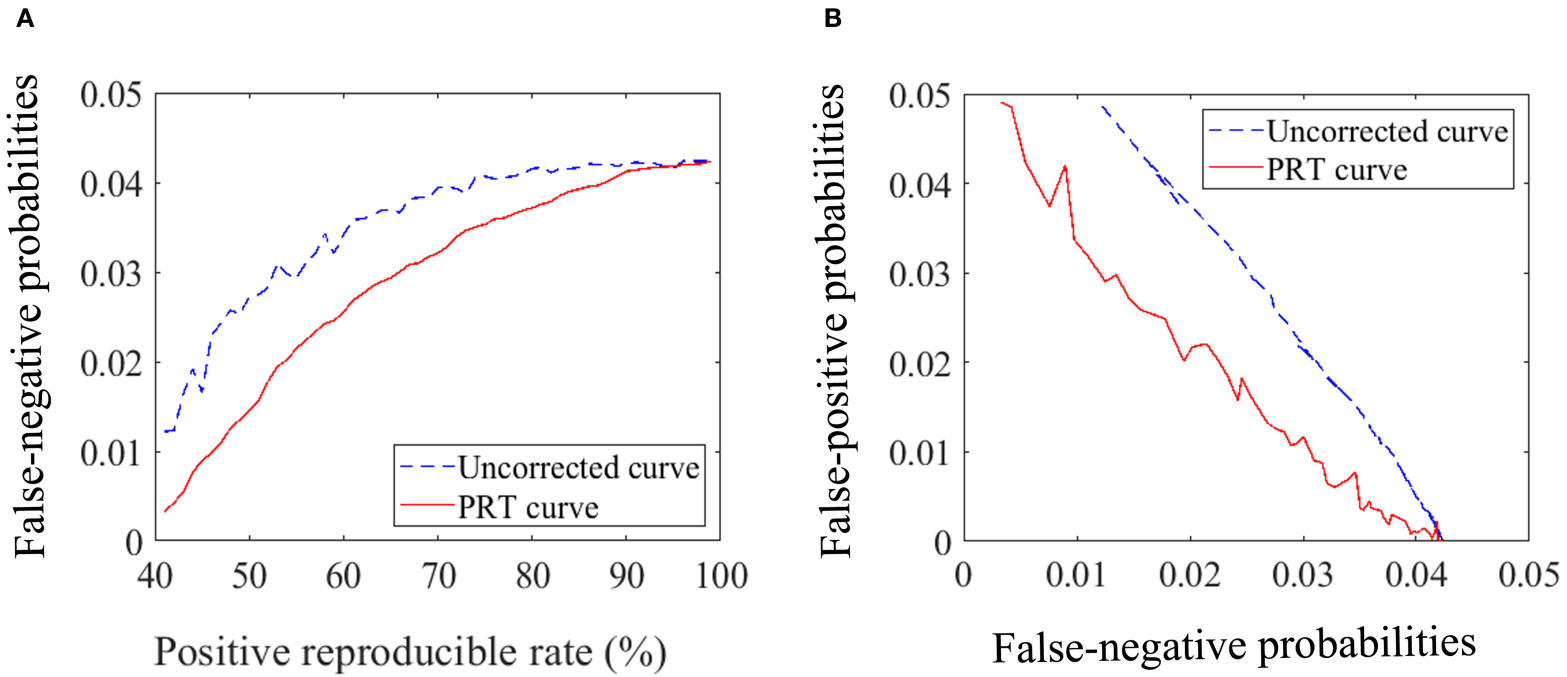
Statistical analysis and comparison. **(A)** Diagram of the relationship between the probability of false negative and the positive reproducible rate. **(B)** Diagram of the relationship between the probability of false positive and negative.

Finally, the statistical analysis and comparison between the PRT correction and the uncorrected are performed, and the results are shown in Figure 1. In Figure 1A, the curves start from the positive reproducibility rate of 41% due to its responding averaged p value is about 0.049 (*p* < 0.05). We analyzed these results and found that the probability of false negatives obtained by PRT correction was lower than that obtained by the uncorrected for the same positive reproducibility rate (i.e., the same probability of false positive) (Figure 1A). Furthermore, we also found that the PRT correction presented lower probability of false positive for the same probability of false negative compared with the uncorrected (Figure 1B). To further verify the validity of the PRT correction, we studied the effect of this method. As shown in Table 3, the PRT correction presented lower false-positive and false-negative probabilities for the same number of positive results compared with the uncorrected.

**Table 3:** Comparison of statistical analysis results for the uncorrected and PRT correction.

**Uncorrected**	**PRT corrected**
(PFP, PFN)	(PFP, PFN)
(0.029055, 0.025729)	(0.015948, 0.025503)
(0.020127, 0.030990723)	(0.009363, 0.030864106)
(0.020432, 0.030994311)	(0.009363, 0.030864106)
(0.012633, 0.035940157)	(0.004472, 0.035885797)
(0.010778, 0.036909172)	(0.003453, 0.036867647)
(0.010877, 0.036909734)	(0.003453, 0.036867647)
(0.006048, 0.039558816)	(0.000804, 0.039543209)
(0.00688, 0.039279796)	(0.001708, 0.039262937)
(0.003484,0.040681022)	(0.001477, 0.040677324)
(0.003536, 0.040681118)	(0.001477, 0.040677324)
(0.002598, 0.041527521)	(0.000802, 0.041525739)
(0.001847, 0.041668237)	(0.000962, 0.041667485)
(0.002573, 0.041527496)	(0.000802, 0.041525739)
(0.002313, 0.041527238)	(0.000802, 0.041525739)
(0.001708, 0.041668119)	(0.000962, 0.041667485)
(0.000804, 0.042092179)	(0.000128, 0.042091891)
(0.001173, 0.041950778)	(0.000061, 0.041950148)
(0.001009, 0.042092266)	(0.000128, 0.042091891)
(0.001477, 0.041809437)	(0.002289, 0.041810012)
(0.000825, 0.042092188)	(0.000128, 0.042091891)
(0.000802, 0.042092178)	(0.000128, 0.042091891)
(0.000962, 0.042092246)	(0.000128, 0.042091891)
(0.002289, 0.041527214)	(0.000802, 0.041525739)
(0.000061, 0.042375292)	(0.000047, 0.04237529)
(0.000128, 0.042375302)	(0.000047, 0.04237529)
(0.000047, 0.04237529)	(0.000047, 0.04237529)

*PFP, probability of false positive; PFN, probability of false negative. The table denotes that comparison of PFP and PFN in the uncorrected and PRT correction for the same number of positive results*.

Our experimental results indicate that higher positive reproducible rate (i.e., “Ri” in the previously published paper (Zhang et al., 2021) means lower probability of false positive rate (i.e., fewer type I errors). In addition, compared with the uncorrected, the PRT correction presented lower false-negative and false-positive probabilities for the same number of positive results.

It is worth noting that selecting higher positive reproducible rate might cause some true positive results to be removed and increase false-negative probabilities.

### Statistical Analyses

Age and sex were analyzed using statistical software (SPSS, version 19.0) to examine whether these demographic characteristics were significantly different. A *p* < 0.05 was regarded as a significant difference.

We selected all BAs as seeds of entropy connectivity and investigated the cross-group differences of entropy connectivity. The PRT correction method was used to correct the results of entropy connectivity. Correction parameters are described as follows. Seventy samples were selected randomly in each group, *p* < 0.05, reproducible rate = 0.85, repeated number of PRT = 1000.

## Results

### Demographic and Behavioral Data Test

A chi-squared test revealed no significant difference in sex between the ASD and TD groups. A two-sample t-test was performed to examine whether there was a significant difference in age across the two groups, and no significant difference was found (Table 4).

**Table 4:** Demographic characteristics of participants.

**Group**	**ASD (*n* = 89)**	**TD (*n* = 94)**	**Statistics (df)**	***P***
Age(ys)	15.1 ± 5.1	16.0 ± 4.2	t = −1.331(181)	0.191
Male/Female	44/45	48/46	X^2^ = 0.048	0.826
Handedness	R	R	–	–
ADOS(ASD)	–	–	–	–
ADOS_TOTAL	11.4 ± 4.1	N/A	N/A	–
ADOS_COMM	3.6 ± 1.7	N/A	N/A	–
ADOS_SOCIAL	7.8 ± 2.9	N/A	N/A	–
ADOS_STEREO_BEHA	1.9 ± 1.6	N/A	N/A	–
IQ(TD)	–	–	–	–
FIQ	N/A	109.2 ± 12.9	–	–
VIQ	N/A	110 ± 13.2	–	–
PIQ	N/A	105.9 ± 14.4	–	–

*Data are in terms of mean ± standard deviation*.

*ASD, autism spectrum disorder; TD, normally developing child; ADOS_TOTAL, Classic Total ADOS Score (Communication subscore + Social Interaction subscore); ADOS_COMM, Communication Total Subscore of the Classic ADOS; ADOS_SOCIAL, Social Total Subscore of the Classic ADOS; ADOS_STEREO_BEHA, Stereotyped Behaviors and Restricted Interests Total Subscore of the Classic ADOS*.

### Cross-Group Differences of Entropy Connectivity From the Prefrontal Cortex to Posterior Brain Regions

**Figure 2:**
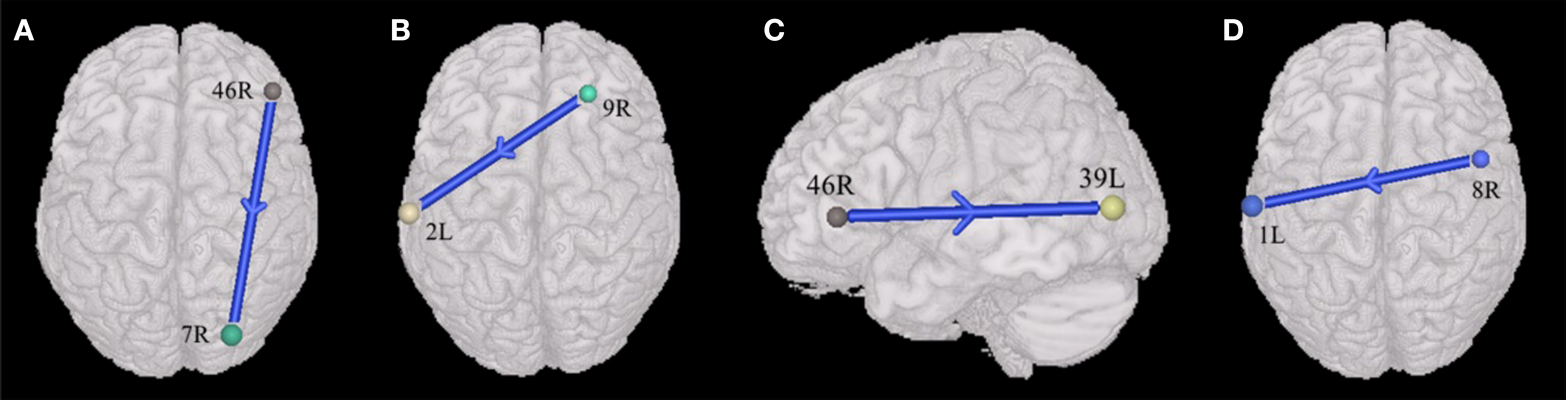
Alterations of the entropy connectivity from the prefrontal cortex to posterior brain regions (*p* < 0.05, two-sided, PRT corrected; reproducible rate: 0.85; number of subjects: 70; PRT threshold: 1,000). **(A)** Synchronous entropy connectivity. **(B–D)** Asynchronous entropy connectivity. The blue arrows denote weakened interregional connections. Every colored sphere in the figure indicates the seed brain region of entropy connectivity, and the color of the sphere is randomly generated and has no special meaning. The number next to the sphere is the index of BA (see Table 2 for details). The direction of the arrow indicates the direction of directional connectivity.

The current study also found that ASDs presented weakened synchronous entropy connectivity from the right dorsolateral prefrontal cortex to the right somatosensory association cortex (BA 46 R → BA 7R) (t = −3.137608, *p* < 0.05, PRT corrected; *p* < 0.003, no corrected) (Figure 2A); weakened asynchronous entropy connectivity from the right dorsolateral prefrontal cortex to the left primary somatosensory cortex (BA 9R → BA 2L) (t = −3.121635, *p* < 0.05, PRT corrected; *p* < 0.003, no corrected) (Figure 2B); weakened asynchronous entropy connectivity from the right dorsolateral prefrontal cortex to the left angular gyrus (BA 46R → BA 39L) (t = −3.25226, *p* < 0.05, PRT corrected; *p* < 0.002, no corrected) (Figure 2C); weakened asynchronous entropy connectivity from the right dorsal frontal cortex to the left primary somatosensory cortex (BA 8 R → BA 1L) (t = −3.583862, *p* < 0.05, PRT corrected; *p* < 0.0005, no corrected) (Figure 2D).

### Cross-Group Differences of Entropy Connectivity From the Posterior Brain Regions to Prefrontal Cortex

**Figure 3:**
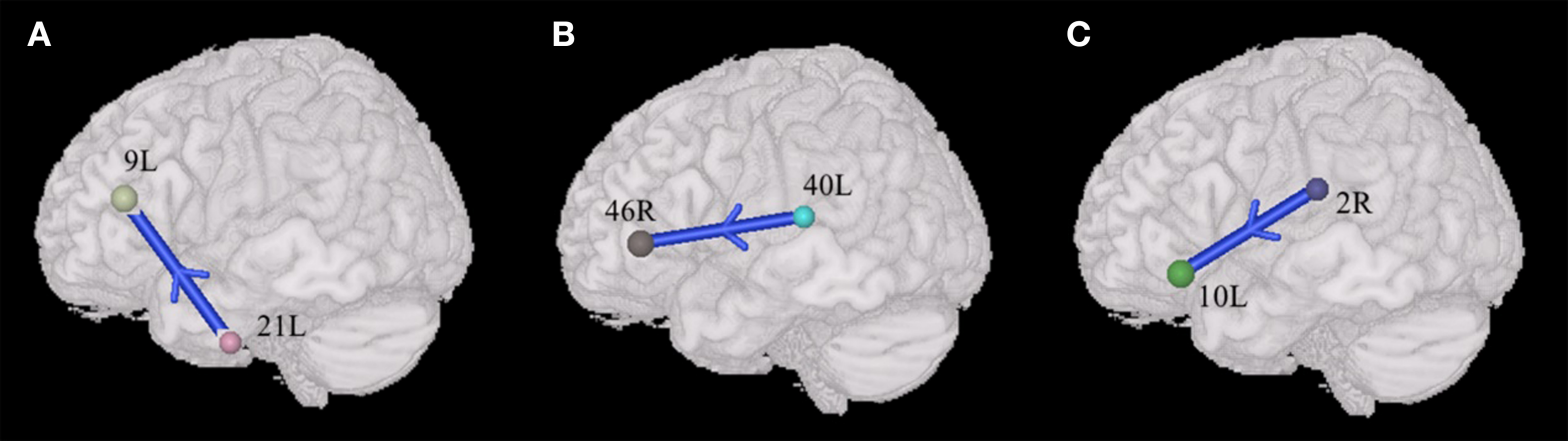
Alterations of the entropy connectivity from the posterior brain regions to prefrontal cortex (*p* < 0.05, two-sided, PRT corrected; reproducible rate: 0.85; number of subjects: 70; PRT threshold: 1000). **(A,B)** Synchronous entropy connectivity. **(C)** Asynchronous entropy connectivity. The blue arrows denote weakened interregional connections. Every colored sphere in the figure indicates the seed brain region of entropy connectivity, and the color of the sphere is randomly generated and has no special meaning. The number next to the sphere is the index of BA (see Table 2 for details). The direction of the arrow indicates the direction of directional connectivity.

Compared with TDs, ASDs represented weakened synchronous entropy connectivity from the left middle temporal gyrus to the left dorsolateral prefrontal cortex (BA 21L → BA 9L) (t = −3.406756, *p* < 0.05, PRT corrected; *p* < 0.0009, no corrected) (Figure 3A); weakened synchronous entropy connectivity from the left supramarginal gyrus to the right dorsolateral prefrontal cortex (BA 40L → BA 46 R) (t = −3.25226, *p* < 0.05, PRT corrected; *p* < 0.002, no corrected) (Figure 3B); weakened asynchronous connectivity from the right primary somatosensory cortex to the anterior prefrontal cortex (BA 2R → BA 10L) (*t* = −3.80959, *p* < 0.05, PRT corrected; *p* < 0.0003, no corrected) (Figure 3C).

### The Differences of Prefrontal-Posterior Connectivity Between ASDs and TDs Within the Male Group

**Figure 4:**
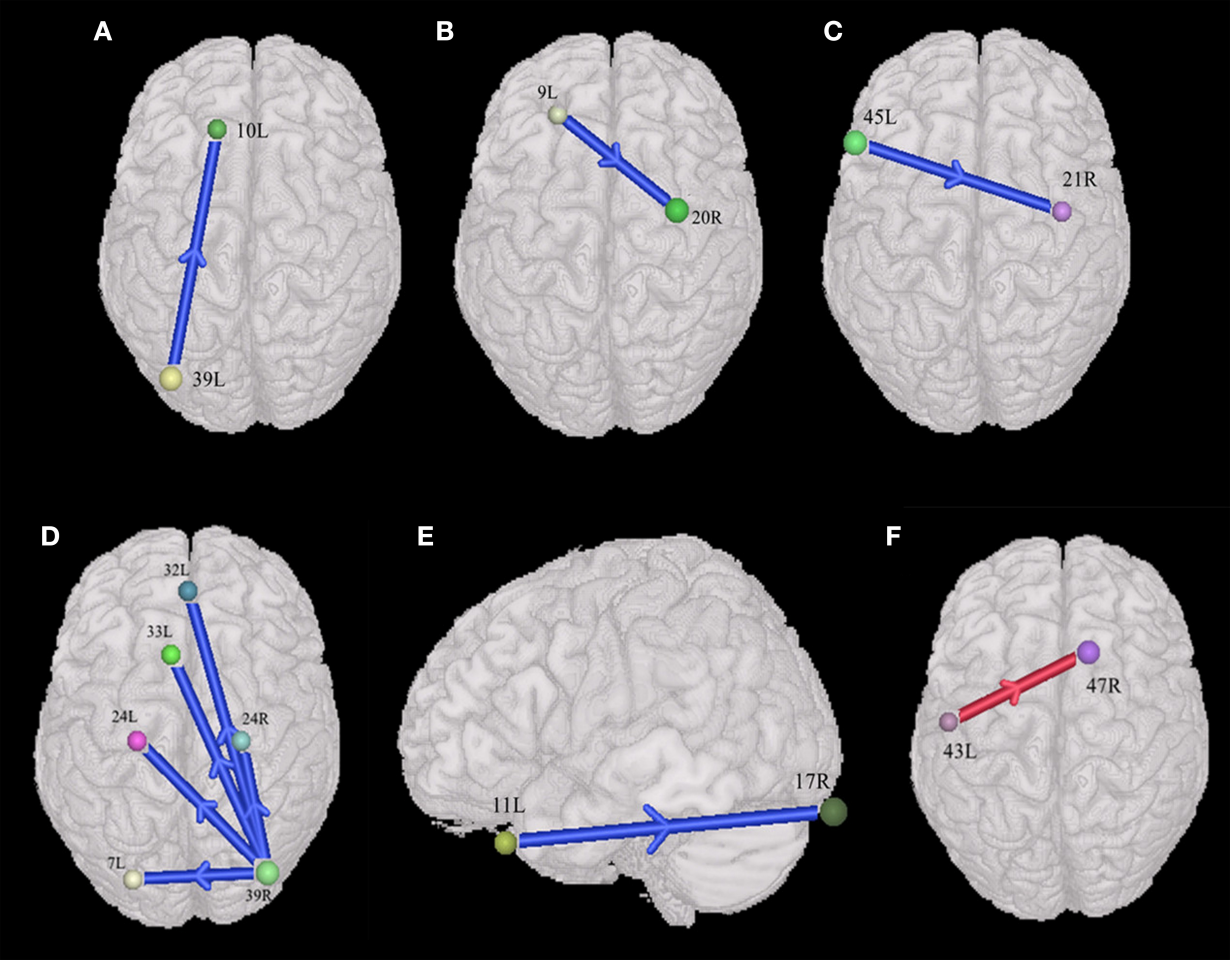
Sex-specific differences of the entropy connectivity in ASD (*p* < 0.05, two-sided, PRT corrected; reproducible rate: 0.85; number of subjects: 30; PRT threshold: 1,000). **(A–E)** Synchronous entropy connectivity in the male group. **(F)** Synchronous entropy connectivity in the female group. The red arrow denotes enhanced interregional connection. The blue arrows denote weakened interregional connections. Every colored sphere in the figure indicates the seed brain region of entropy connectivity, and the color of the sphere is randomly generated and has no special meaning. The number next to the sphere is the index of BA (see Table 2 for details). The direction of the arrow indicates the direction of directional connectivity.

In the male group (45 males with ASD vs. 46 males with TD), the males with ASD presented the weakened synchronous entropy connectivity from the left angular gyrus to the left anterior prefrontal cortex (BA 39L → BA 10L) (t = −3.768572, *p* < 0.05, PRT corrected; *p* < 0.0003, no corrected) (Figure 4A); weakened synchronous entropy connectivity from the left dorsolateral prefrontal cortex to the right inferior temporal gyrus (BA 9L → BA 20R) (t = −4.194185, *p* < 0.05, PRT corrected; *p* < 0.00008, no corrected) (Figure 4B); weakened synchronous entropy connectivity from the left IFC pars triangularis to the right middle temporal gyrus (BA 45L → BA 21R) (t = −3.785974, *p* < 0.05, PRT corrected; *p* < 0.0003, no corrected) (Figure 4C); weakened synchronous entropy connectivity from the right angular gyrus to the left dorsal cingulate cortex (BA 39R → BA 32L) (t = −3.81218, *p* < 0.05, PRT corrected; *p* < 0.0003, no corrected) (Figure 4D); weakened synchronous entropy connectivity from the right angular gyrus to the left anterior cingulate cortex (t = −3.316281, *p* < 0.05, PRT corrected; *p* < 0.002, no corrected) and weakened synchronous entropy connectivity from the right angular gyrus to the bilateral ventral anterior cingulate cortex (BA 39R → BA 24L) (t = −3.649195, *p* < 0.05, PRT corrected; *p* < 0.0008, no corrected) (BA 39R → BA 24R) (t = −3.981538, *p* < 0.05, PRT corrected; *p* < 0.0003, no corrected) (Figure 4D); weakened synchronous entropy connectivity from the left orbitofrontal cortex to the right primary visual cortex (BA 11L → BA 17R) (t = −3.718285, *p* < 0.05, PRT corrected; *p* < 0.0005, no corrected) (Figure 4E). In addition, there was an atypical connectivity between the right angular gyrus and the left somatosensory association cortex, but it was not the prefrontal-posterior underconnectivity in this study (BA 39R → BA 7L) (t = −3.958346, *p* < 0.05, PRT corrected; *p* < 0.0002, no corrected) (Figure 4D).

### The Differences of Prefrontal-Posterior Connectivity Between ASDs and TDs Within the Female Group

In the female group (44 females with ASD vs. 48 females with TD), we only found the enhanced synchronous entropy connectivity from the left subcentral area to the right inferior prefrontal gyrus (BA 43L → BA 47L) (t = 3.672919, *p* < 0.05, PRT corrected; *p* < 0.008, no corrected) (Figure 4F).

## Discussion

We used resting-state functional imaging of the ASD and TD groups to construct entropy connectivity and examined differences in functional connectivity between the prefrontal and posterior brain regions across both the ASD and TD groups. Specifically, in the present study, we found that ASDs presented weakened entropy connectivity between the prefrontal cortex (BAs 8R, 9L, 9R, 10L, 46R) and the primary somatosensory cortex (BAs 1L, 2L, 2R), somatosensory association cortex (BA 7R), middle temporal gyrus (BA 21L), angular gyrus (BA 39L), and supramarginal gyrus (BA 40L). In addition, we also discussed interactive prefrontal-posterior underconnectivity based on the predictive coding theory.

### The Impaired Information Flow From the Prefrontal Cortex to the Posterior Brain Regions in ASD

To limit cognitive resources of maintaining context information (Braver and Cohen, 2001) and filter unnecessary information (Jun and Hoshi, 2008), as well as top-down attention (Buschman and Miller, 2007), the neural activities of the posterior brain areas are often accepted from the modulation of high-level brain regions, such as the prefrontal cortex. Some studies have reported that the absence of the active guidance mediated by the prefrontal cortex in individuals with ASD might have a negative impact on early sensory processing, execution function (Frith, 2008) and face recognition (Bird et al., 2006).

Abnormal functional connectivity between the prefrontal lobe (a higher-order brain region) and the primary somatosensory cortex (a lower-order brain region) has been found in some ASD studies. A previous study reported that children with ASD presented increased functional connectivity between the primary sensory and association regions (including the lateral frontal and parietal cortices) (Supekar et al., 2013). In the current study, we found decreased asynchronous entropy connectivity from the right dorsal prefrontal cortex (BAs 8R, 9R) to the left somatosensory cortex (BAs 1L, 2L), and the prefrontal cortex has been proven to play an important role in regulating information processing of the primary sensory cortex (Staines et al., 2002). A previous investigation found that patients with DLPFC injury displayed destroyed inhibitory regulation of inputs to the primary somatosensory cortex, and weakened inhibitory regulation often led to weakened processing for task-irrelevant sensory signals (Yamaguchi and Knight, 1990; Robert et al., 1999). Therefore, the weakened asynchronous entropy connectivity from the dorsal prefrontal to somatosensory area in ASD may indicate decreased selective collection of sensory information guided by the prefrontal cortex (restraining meaningless input and deciding relevant input) and further lead to decoupling between cognitive processes and sensory information from the environment (Miller and Cohen, 2001), which manifests as atypical somatosensory processing (Sapey-Triomphe et al., 2019).

Just and his colleagues (Koshino et al., 2005) found that the autistic patients with IQs in the normal range presented decreased functional connectivity between the right dorsolateral prefrontal lobe and the left inferior parietal lobule during an n-back visual working memory task, which was considered to be an important reason that ASDs need to rely more on the posterior brain region for information processing in visual tasks. In the current study based on resting-state fMRI, we also found that the right dorsolateral prefrontal lobe (BA 46R) in ASD displayed weakened synchronous output entropy connectivity to the ipsilateral parietal lobule (BA 7R) but asynchronous output entropy connectivity to the contralateral angular gyrus (BA 39L), and previous literature has reported that BA 7R is involved in the mental manipulation of information in working memory (Koenigs et al., 2009), the coordination of visual–tactile conflict (Ro et al., 2004) and visual-spatial processing (Ro et al., 2004). Additionally, BA 39L participates in multiple cognitive processes such as semantic processing, visual spatial processing, the manipulation of mental representations and memory retrieval (Seghier, 2013). Thus, these results might potentially affect the active maintenance of the prefrontal cortex for the information in the multimodal cortical architectures during “rest.”

### Autism Declined Capacity in Information Flow From the Posterior Brain Regions to the Prefrontal Cortex

The current study found weakened synchronous entropy connectivity from the middle temporal gyrus (BA 21L) to the dorsolateral prefrontal cortex (BA 9L), and the middle temporal gyrus plays an important role in multimodal visual information processing (Hidaka et al., 2015; Stock et al., 2017). In a study based on resting-state functional connectivity, an investigator found that insufficient functional correlation between the left anterior middle temporal gyrus and the right frontal polar cortex affected superior cognitive brain functions in children with ASD (Borras-Ferris et al., 2019).

Additionally, we also observed weakened synchronous entropy connectivity from the supramarginal gyrus (BA 40L) to the dorsolateral prefrontal cortex (BA 46R). BA 40 is a part of the inferior parietal lobe (IPL) (Caspers et al., 2006), and IPL is believed to integrate multisensory information and participate in various high-level cognitive activities, such as executive function and self-awareness (Torrey, 2007). It is believed that in executive function-related tasks, bandwidth limitations in the frontal and parietal lobes might lead to the disrupted information coordination between two regions, which is associated with cognitive deficits in ASD (Just et al., 2012). Furthermore, a study suggested that the lateral prefrontal cortex (LPFC) participates in integrating highly processed cognitive and motivational information from the posterior association cortices (e.g., IPL, middle temporal gyrus) and the orbitofrontal cortex, respectively, for adaptive goal-directed behavior (Watanabe and Sakagami, 2007). Therefore, we proposed that the weakened synchronous entropy connectivity mentioned above might affect the integrity of cognitive information input from the posterior brain regions to the prefrontal cortex.

Interestingly, we also found weakened asynchronous entropy connectivity between the primary somatosensory cortex (BA 2R) and the anterior prefrontal cortex (BA 10L). Peng et al. (2018) proposed that the anterior prefrontal cortex might participate in the integration and advanced processing of nociception and pain, and in this process, the BA 10 can integrate the sensory aspect of pain by the information flow from the sensorimotor network to the lateral BA 10. Some studies also presented that BA 10 contributes to the process of transforming tactile and somatosensory information into abstract representation to maintain hapticospatial information during cognitive tasks (Kaas et al., 2007; Matsumoto et al., 2020). Thus, weakened entropy connectivity may potentially limit sensory information integration between brain areas in ASD.

### The Interactive Prefrontal-Posterior Underconnectivity From the Perspective of Predictive Coding Theory

**Figure 5:**
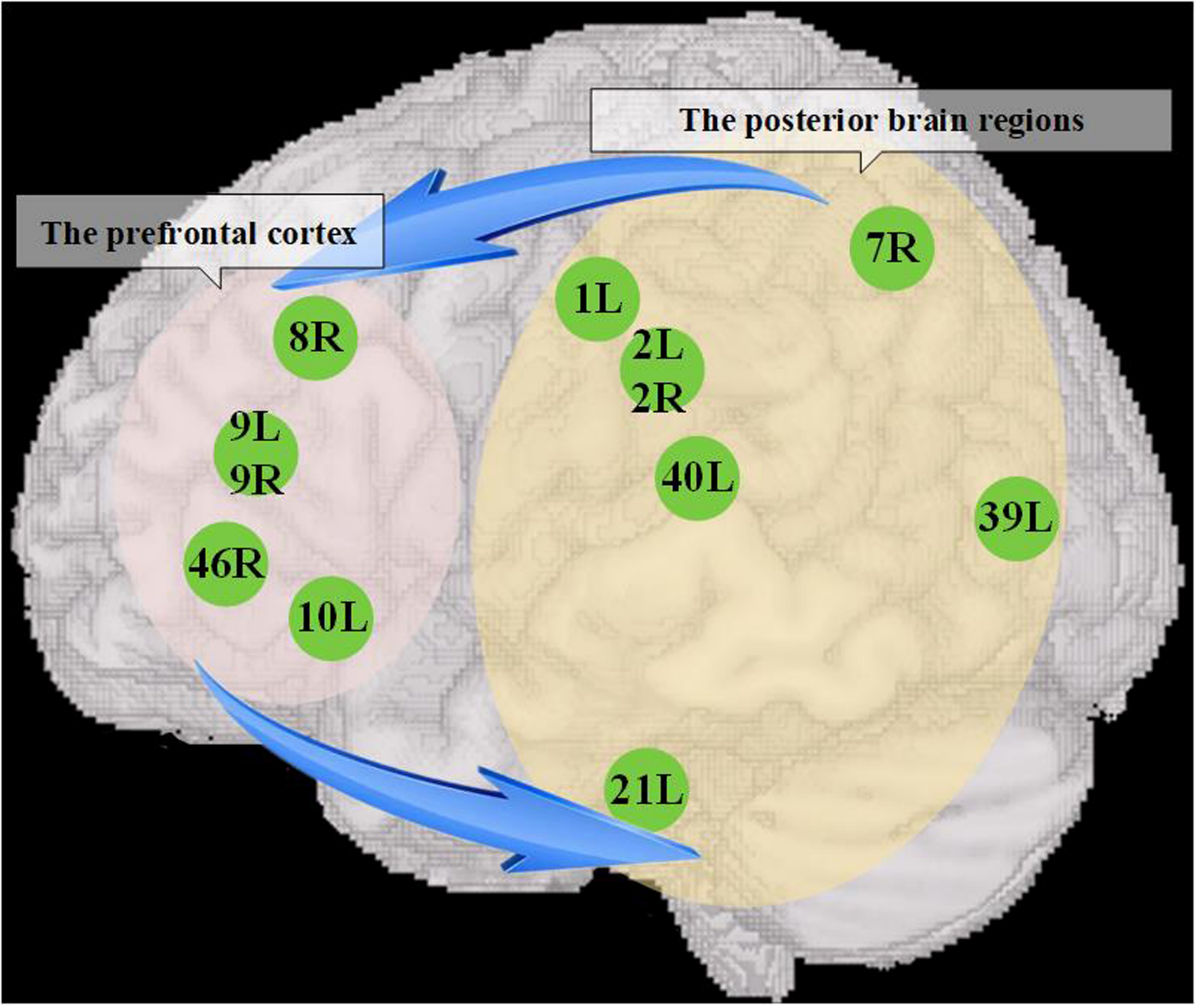
The dysfunction of interactive information processing between the prefrontal cortex and posterior brain regions. The left elliptical shadow area covers the prefrontal cortex, and the right elliptical shadow area covers the posterior brain regions, and the figures in the shadow area show the seed brain regions with atypical entropy connectivity found in this study. The blue arrow represents that there might be a reduced mutual information transmission between them, which further contributed to accelerate the deoptimization of brain networks.

By using a method of directional connectivity, entropy connectivity, our results provide direct evidence that there is a functional interactive underconnectivity pattern between the prefrontal cortex and posterior brain areas. Notably, based on the previous discussion, we can summarize the pattern as the dysfunction of the interactive information processing between two brain regions, which consists of two weakened information pathways, i.e., the information flow from the prefrontal cortex to posterior brain regions and the information flow from the posterior brain regions to prefrontal cortex (Figure 5). Our suppositions are based on predictive coding theory, which constructs a framework that states that the brain is not a coded stimulus-response machine but a statistical organ that actively interprets the stimuli it encounters, tested on sensory evidence (Seth and Friston, 2016). Specifically, the brain builds prior knowledge to produce prediction signals, which are integrated with sensory signals to understand the external world, but if there is mismatching between two kinds of signals, the prediction error signals are uploaded to higher hierarchical structures to update prior knowledge to make the internal representation more compatible with the external environment or change the sensory information to make them more like predictions (Seth and Friston, 2016; Brodski-Guerniero et al., 2018; Coll et al., 2020). Notably, Smith et al. (2017) suggested that the prefrontal cortex possessing multimodal prior knowledge sends the downward high-precision estimates to other brain regions to promote goal-directed thought, and it accepts the modification to its prior knowledge from salient prediction error information broadcasted by other brain structures. According to the aforementioned theory, the weakened connectivity from the prefrontal cortex to posterior brain regions might imply the impairment of the cognitive control in ASDs. That is, the prefrontal cortex cannot flexibly allocate mental resources by adjusting the flow of information in the posterior brain areas to guide thoughts and actions in light of prior knowledge (Solomon et al., 2009). Inhibited connectivity from the posterior brain regions to prefrontal cortex might impair the process by which the prefrontal cortex (especially the DLPFC) recodes perception information from posterior cortical areas to an abstract form Wicker et al. (2008) to effectively store and control the information. On the other hand, the lack of some prediction error signals endowed with high precision to privilege access to the prefrontal cortex will make it difficult to update prior knowledge (Seth and Friston, 2016). Taken together, the interactive prefrontal-posterior underconnectivity may induce the deoptimization of the autistic brain networks due to potentially affecting the information processing pattern of the brain that integrating the information from the body and environment with prior knowledge (Friston, 2010; Sterling, 2012).

However, many studies agreed that patients with ASD have abnormal prediction signals and relatively complete and even enhanced prediction error signals (Maekawa et al., 2011; Brodski-Guerniero et al., 2018) from the perspective of predictive coding theory. We believe that one of the reasons for this conflict is a difference in approach: Maekawa et al. (2011) and Brodski-Guerniero et al. (2018) used EEG and MEG, respectively, to examine the changes in brain function in ASD. The directional functional connectivity we use is more visualized to reflect the direction of neurodynamics. An EEG study using the hierarchical frequency tagging task identified atypical integration of prediction and prediction error signals in ASD. Specifically, atypical precise weighted integration (IM component) of both prediction and prediction error signals was associated with lower ASD characteristics, while such changes were not found in patients with higher ASD characteristics (Coll et al., 2020). We proposed that the changes in interactive information processing in ASD would further worsen brain network optimization, and the symptoms of ASD might be more serious. This may explain the atypical interactive information processing found in the abovementioned study at lower rather than higher ASD characteristics.

In addition, we also found the enhanced asynchronous entropy connectivity between right dorsal frontal cortex (BA 8R) and left piriform cortex (BA 27L) (t = 3.199877, *p* < 0.05, PRT corrected; *p* < 0.002, no corrected). Many researchers have confirmed that the functional connectivity of the patients with ASD presented under- and over-connectivity simultaneously (Hull et al., 2017). This result may indicate the heterogeneity of autistic functional connectivity. However, the reason why our study did not discuss this enhanced asynchronous connectivity was that the piriform cortex is the paleocortex rather than the posterior brain areas discussed in this study (De Curtis et al., 2019). Therefore, we could obtain a consistent conclusion: there is the interactive underconnectivity between the prefrontal cortex and posterior brain regions.

### Comparison Between the Results Using ComBat and the Original

**Figure 6:**
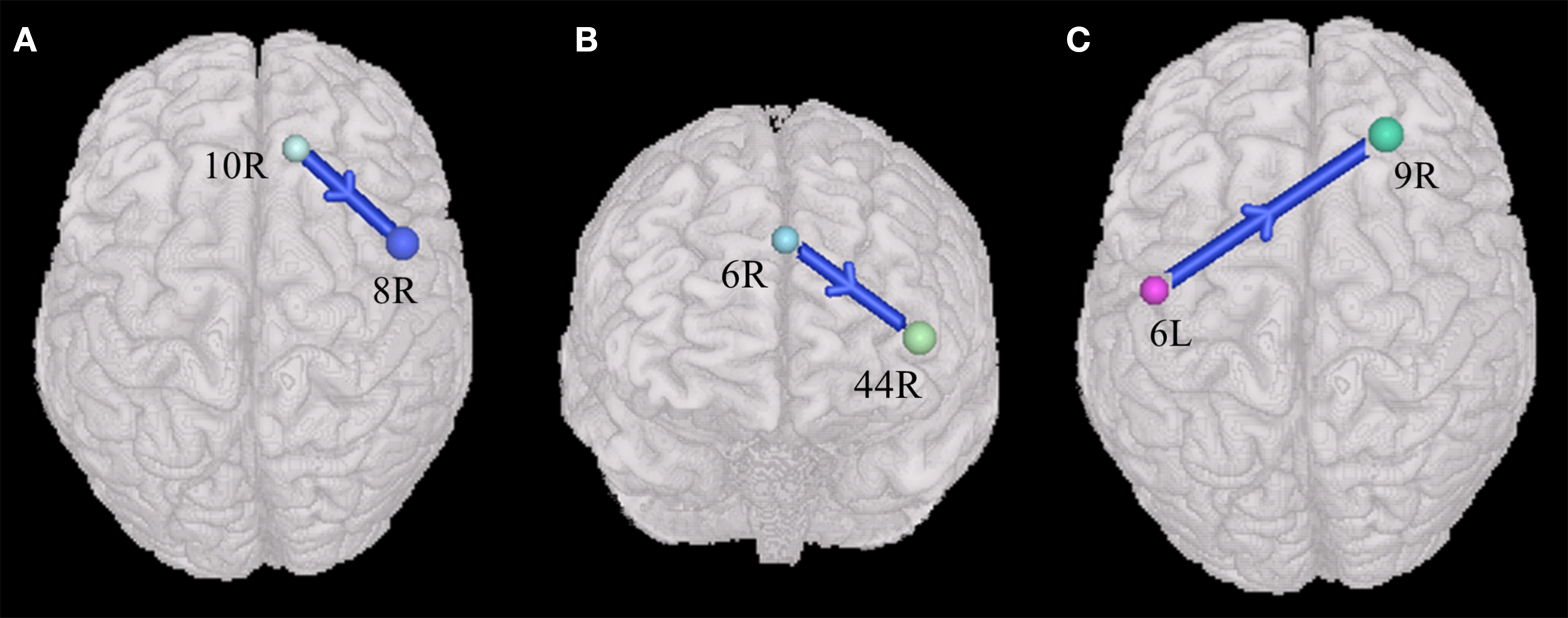
Weakened synchronous entropy connectivity (BA 21L → BA 10L) (*p* < 0.05, two-sided, PRT corrected; reproducible rate: 0.85; number of subjects: 70; PRT threshold: 1,000). The blue arrow denotes weakened interregional connectivity. Every colored sphere in the figure indicates the seed brain region of entropy connectivity, and the color of the sphere is randomly generated and has no special meaning. The number next to the sphere is the index of BA (see Table 2 for details). The direction of the arrow indicates the direction of directional connectivity.

There was some consistent results between the prefrontal cortex and posterior brain regions in these two experiments with or without ComBat harmonization (*p* < 0.05, reproducible rate: 0.85, PRT corrected). Only those with significant entropy connectivity between the prefrontal cortex and posterior brain regions are described as follows. Weakened synchronous entropy connectivity from the right dorsolateral prefrontal cortex to the right somatosensory association cortex (BA 46R → BA 7R) (*t* = −3.360816, *p* < 0.05, PRT corrected; *p* < 0.002, no corrected); weakened asynchronous connectivity from the right primary somatosensory cortex to the anterior prefrontal cortex (BA 2R → BA 10L) (t = −3.57506, *p* < 0.05, PRT corrected; *p* < 0.0005, no corrected); weakened asynchronous entropy connectivity from the right dorsal frontal cortex to the left primary somatosensory cortex (BA 8R → BA 1L) (t = −3.706519, *p* < 0.05, PRT corrected; *p* < 0.0004, no corrected); weakened asynchronous entropy connectivity from the right dorsolateral prefrontal cortex to the left angular gyrus (BA 46R → BA 39L) (t = −3.214031, *p* < 0.05, PRT corrected; *p* < 0.002, no corrected). In addition, we also found weakened synchronous entropy connectivity from the left middle temporal gyrus to the left anterior prefrontal cortex (BA 21L → BA 10L) (t = −3.827444, *p* < 0.05, PRT corrected; *p* < 0.0002, no corrected), which is a newly appearing result in the prefrontal cortex and the posterior brain region (Figure 6).

Although there are some differences between the ComBat result and the original, we obtain a consistent conclusion: there exists interactive underconnectivity between the prefrontal cortex and posterior brain regions on the basis of these two results.

### Sex-Specific Differences of the Entropy Connectivity in ASD

Many previous studies provided much evidence of sex bias in autism from the perspective of gene, testosterone, immune system, and functional connectivity (Hu et al., 2015; Lai et al., 2017; Ferri et al., 2018; Cummings et al., 2020). However, it remains underaddressed whether there exist sex-specific differences in the directional connectivity network. In this present study, we investigated the differences of the entropy connectivity between ASDs and TDs within the female and male groups respectively. We found that the autistic males presented the interactive prefrontal-posterior underconnectivity compared with the males with TD (Figures 4A–E), but there existed an overconnectivity from the posterior cortex to prefrontal cortex between ASDs and TDs in the female (Figure 4F).

Alaerts et al. (2016) reported that the males with ASD generally presented underconnectivity but the females with ASD generally exhibited overconnectivity relative to sex-matched TDs in intrinsic functional connectivity, and they suggested the sex-specific differences of functional connectivity might be related to the disturbances in sex steroid levels (e.g., fetal testosterone). Thus, the aforementioned results indicated that the interactive prefrontal-posterior underconnectivity cannot necessarily be generalized to females with autism. We also suggested that future studies with larger samples of the males and females may help reveal the potential mechanism of the sex-specific differences of ASD in directional connectivity network.

## Conclusions

In this article, we investigated alterations in entropy connectivity between the prefrontal cortex and posterior brain regions. Resting-state neural activity in individuals with ASD presented interactive prefrontal-posterior underconnectivity. We suggested that interactive prefrontal-posterior underconnectivity might lead to less prior knowledge being used and updated from the perspective of predictive coding theory. Our proposals highlight that a combination of impaired interactive information flow between the prefrontal cortex and posterior brain regions accelerates the deoptimization of brain networks of ASD. Last, by analyzing the sex-specific differences of the entropy connectivity, we found this underconnectivity cannot necessarily be generalized to females with autism.

## Limitations

There are several limitations of this study that warrant discussion. First, we limited the variability of handedness and the sex ratio of the data and removed unavailable data that were of poor imaging quality and were acquired from the patients with ASD using psychoactive drugs. We found no significant difference between the ASD and TD groups regarding age. To expand the data volume, we relatively loosened the treatment of the control about sites, resulting in variability of image data scanning parameters, which is consistent with the original intention of the database builders (Di Martino et al., 2014). Second, we did not investigate a dimensional associations with ASD symptoms due to the lack of clinical test scores (e.g., ADOS scores) in twenty-nine ASDs and eighty-four TDs. Moreover, some researchers proposed that the brain network of children with ASD under 12 years old showed overconnectivity compared with TD and the adolescents and adults with ASD appeared weakened functional connectivity (Uddin et al., 2013), which was speculated to be related to the abnormally accelerated growth of white matter in early children and subsequent loss of white matter in adolescence and adulthood (Waiter et al., 2005; Maximo et al., 2014). Therefore, we suggested that the finding of this study (i.e., the interactive prefrontal-posterior underconnectivity) only applicable to autistic adolescents and young males (15 ± 5 years) with IQs in the normal range. Developmental studies will be need to determine how these alterations in brain networks arise.

## Data Availability Statement

The datasets presented in this study can be found in online repositories. The names of the repository/repositories and accession number(s) can be found below: http://fcon_1000.projects.nitrc.org/indi/abide/abide_I.html.

## Ethics Statement

The studies involving human participants were reviewed and approved by the Institutional Ethics Committee of Shandong First Medical University. Written informed consent from the participants' legal guardian/next of kin was not required to participate in this study in accordance with the national legislation and the institutional requirements. Written informed consent was not obtained from the individual(s), nor the minor(s)' legal guardian/next of kin, for the publication of any potentially identifiable images or data included in this article.

## Author Contributions

RL and H-CZ conceived and designed the study. X-TC, RL, and G-YZ contributed to experimental design. H-CZ, RL, and G-YZ performed the experiments. H-CZ, QS, and RL wrote the first draft of the manuscript. X-TC, RL, H-CZ, L-MH, QS, and G-YZ discussed results. G-YZ, X-YB, X-YL, and C-YY revised the first draft of the manuscript. All authors contributed to the revision of the final version of the manuscript, read and approved the submitted version.

## Funding

This research was supported by the Shandong Students' Platform for Innovation and Entrepreneurship Training Program (S202110439094), Shandong Provincial Natural Science Foundation, China (Grant Number: ZR2018MH033, ZR2015HL095, and ZR2014HM072), the High-Level Project Nurturing Program of Taishan Medical University (2018GCC13), the Shandong Province Higher Educational Science and Technology Program (No. J17KA262), and the Academic Promotion Program of Shandong First Medical University (2019LJ004).

## Conflict of Interest

The authors declare that the research was conducted in the absence of any commercial or financial relationships that could be construed as a potential conflict of interest.

## Publisher's Note

All claims expressed in this article are solely those of the authors and do not necessarily represent those of their affiliated organizations, or those of the publisher, the editors and the reviewers. Any product that may be evaluated in this article, or claim that may be made by its manufacturer, is not guaranteed or endorsed by the publisher.

## Abbreviations

ASD: autism spectrum disorder
ABIDE I: Autism Brain Imaging Data Exchange I
BA: Brodmann area
CDC: Centers for Disease Control and Prevention
DCM: dynamic causal modeling
DLPFC: dorsolateral prefrontal cortex
DPARSF: Data Processing Assistant for Resting-State fMRI
EEG: electroencephalography
fMRI: functional magnetic resonance imaging
IPL: inferior parietal lobe
IQ: intelligence quotient
LPFC: lateral prefrontal cortex
MEG: magnetoencephalography
PFC: prefrontal cortex
PRT: positive reproduce test
TD: typical developing.
